# Direct detection of phycocyanin in sediments by hyperspectral imaging

**DOI:** 10.1007/s10933-024-00350-y

**Published:** 2024-12-30

**Authors:** Giulia Wienhues, Petra Zahajská, Daniela Fischer, Tobias Schneider, Martin Grosjean

**Affiliations:** 1 Institute of Geography and Oeschger Center for Climate Change Research, University of Bern, Hallerstrasse 12, 3012 Bern, Switzerland; 2 Institute of Geology and Palaeontology, Faculty of Science, Charles University, Albertov 6, Prague, 12843 Czechia; 3 Department of Surface Waters–Research and Management, Swiss Federal Institute of Aquatic Science and Technology (EAWAG), Überlandstrasse 133, 8600 Dübendorf, Switzerland

**Keywords:** Cyanobacteria, Algal blooms, Pigments, Paleolimnology, Environmental change

## Abstract

Cyanobacteria are ubiquitous aquatic organisms with a remarkable evolutionary history reaching as far as 1.9 Ga. They play a vital role in ecosystems yet also raise concerns due to their association with harmful algal blooms. Understanding the historical patterns and drivers behind these blooms is crucial for effective ecosystem management. Lake-sediment cores are valuable natural environmental archives, recording the histories of such blooms. Among others, phycocyanin, a pigment specific to cyanobacteria, emerges as a promising biomarker for reconstructing past cyanobacterial bloom events. However, due to the physicochemical properties of phycocyanin, there is no validated method available to extract and measure this pigment from complex sediment matrix. This study explores the applicability of hyperspectral imaging (HSI), a non-destructive technique, as a novel approach for high resolution *in-situ* detection and quantification of phycocyanin in lake sediments. Our experiments show that phycocyanin can be detected by HSI with an absorption trough at 620 nm (relative absorption band depth, RABD_620_). We established a semi-quantitative calibration of the spectral index RABD_620_ by conducting spiking experiments with phycocyanin standard (known phycocyanin mass) on organic-rich and mineral-rich sediments of varying water contents. We also assessed potential interference from chlorophyll *a*, another photosynthetic pigment, ensuring the reliability of hyperspectral phycocyanin measurements. Our findings demonstrate a significant correlation (R^2^ ranging from 0.37 to 0.997) between the RABD_620_ index and associated phycocyanin amounts in organic-rich and minerogenic sediments. This indicates the potential of the spectral index to directly measure *in-situ* biomarker concentrations on split sediment cores. Although confounding factors such as water and chlorophyll *a* content can influence the spectral signal, this method offers a rapid and non-destructive approach for studying historical cyanobacterial blooms in sedimentary records. This opens promising grounds for various applications, including ecosystem-health assessment and environmental change monitoring.

**Supplementary Information:**

The online version contains supplementary material available at 10.1007/s10933-024-00350-y.

## Introduction

Eutrophication, a global water-quality concern, harms aquatic ecosystems by degrading water quality, reducing biodiversity, and prompting harmful algal blooms, often dominated by cyanobacteria (O’Neil et al. 2012). Factors such as nutrient enrichment, thermal stratification, and hypoxia favour cyanobacteria dominance (Carey et al. 2012). Monitoring studies suggest that human-induced eutrophication amplifies the frequency and intensity of toxic cyanobacteria blooms. Such blooms have occurred for centuries in aquatic ecosystems, but little is known about the natural baselines, as studies mostly focus on modern lake systems.

Phycocyanin (PC), a pigment specific to cyanobacteria, emerges as a promising biomarker for estimating cyanobacteria abundance with remote sensing techniques (Li et al. 2010; Le et al. 2011; Sun et al. 2013). Recent studies show that correlations between PC and cyanobacterial biomass generally have high coefficients of determination (Randolph et al. 2008; Horváth et al. 2013), highlighting that PC is a reliable proxy to estimate cyanobacteria biomass in surface waters.

Lake sediments are ideal environmental archives to study algal productivity beyond the observational period (Michelutti and Smol 2016), potentially including past cyanobacterial blooms through PC. However, due to PC’s chemical properties and solubility, the traditional organic solvent-based sedimentary pigment extraction methods such as Lami et al. (1994) or Reuss (2005), are not suitable for PC. Moreover, the predominantly used extraction workflows for PC focus on cyanobacteria cultures or water samples (Doke 2005; Benedetti et al. 2006; Moraes et al. 2011; İlter et al. 2018; Li et al. 2020), and their applicability to sedimentary PC is understudied.

The PC pigment is part of the major accessory pigment group in cyanobacteria, the phycobiliproteins (Benedetti et al. 2006). Phycobiliproteins are arranged in a complex structure—phycobilisomes, which form the light harvesting complex of cyanobacteria. They reside in antenna-like structures surrounding photosynthetic reaction centres (Padyana et al. 2001; Jaeschke et al. 2021). The blue, water-soluble PC is a phycobiliprotein composed of two sub-units covalently bound with chromophore called phycocyanobilin (Jaeschke et al. 2021). The colour of PC stems from the chromophore phycocyanobilin, which absorbs light between 540 and 620 nm (*A*_*λ*_ = 620 nm) (Hsieh-Lo et al. 2019). This unique protein structure of phycobiliproteins increases the stability and protects the phycocyanobilin (Jaeschke et al. 2021).

Thus, to extract PC, the cell membrane must be disintegrated, and phycobilisomes must be dissolved in buffer solution. PC is commonly extracted from the algal biomass by leaching in distilled water or sodium phosphate buffer of pH 6.8 and separating it from the protein complex by various reagents such as sodium dodecyl sulphate or urea (Sarada et al. 1999). However, the covalent bond between the chromophore and the protective protein structure is affected by temperature, pH, solvent type and method of cell-wall disintegration (Lawrenz et al. 2011; Zimba 2012; Jaeschke et al. 2021) and, thus, the PC extraction often results in low purity extracts with low stability of the pigment.

To date, very little information is known on the form of PC in sediments and soils. To our best knowledge, no method exists for PC extraction from sediments or soils. Preliminary tests (this study Material and methods: *Preliminary extraction tests*) reveal the significant challenge of desorbing phycobiliproteins, including PC, from the sediment matrix. Therefore, *in-situ* detection of PC in sediments by non-destructive hyperspectral imaging, avoiding the extraction challenges, becomes a promising tool for direct detection of the coloured cyanobacterial pigment PC in lake sediments and bacterial mats (Mehrubeoglu et al. 2012, 2013; Sorrel et al. 2021; Zander et al. 2022). Noteworthy is the Holocene PC record inferred from hyperspectral imaging (absorption band at 615 nm) for a lake in Kyrgyzstan (Sorrel et al. 2021). These authors were, however, not able to ground truth and verify the relation of the RABA_615_ to the presence of PC.

Our study explores the potential of PC detection and quantification in lake sediments as a cyanobacteria biomarker using hyperspectral imaging. We employ a spiking experiment to test the absorption behaviour of PC on sediment samples with varying organic matter, water, and chlorophyll *a* (Chl *a*) content. We demonstrate that (i) PC exhibits a specific absorption feature at 620 nm in sediments and that (ii) absorption features are proportional to the PC concentration/mass, enabling quantification of relative changes in PC concentration using scanning hyperspectral imaging.

## Material and methods

**Fig. 1:**
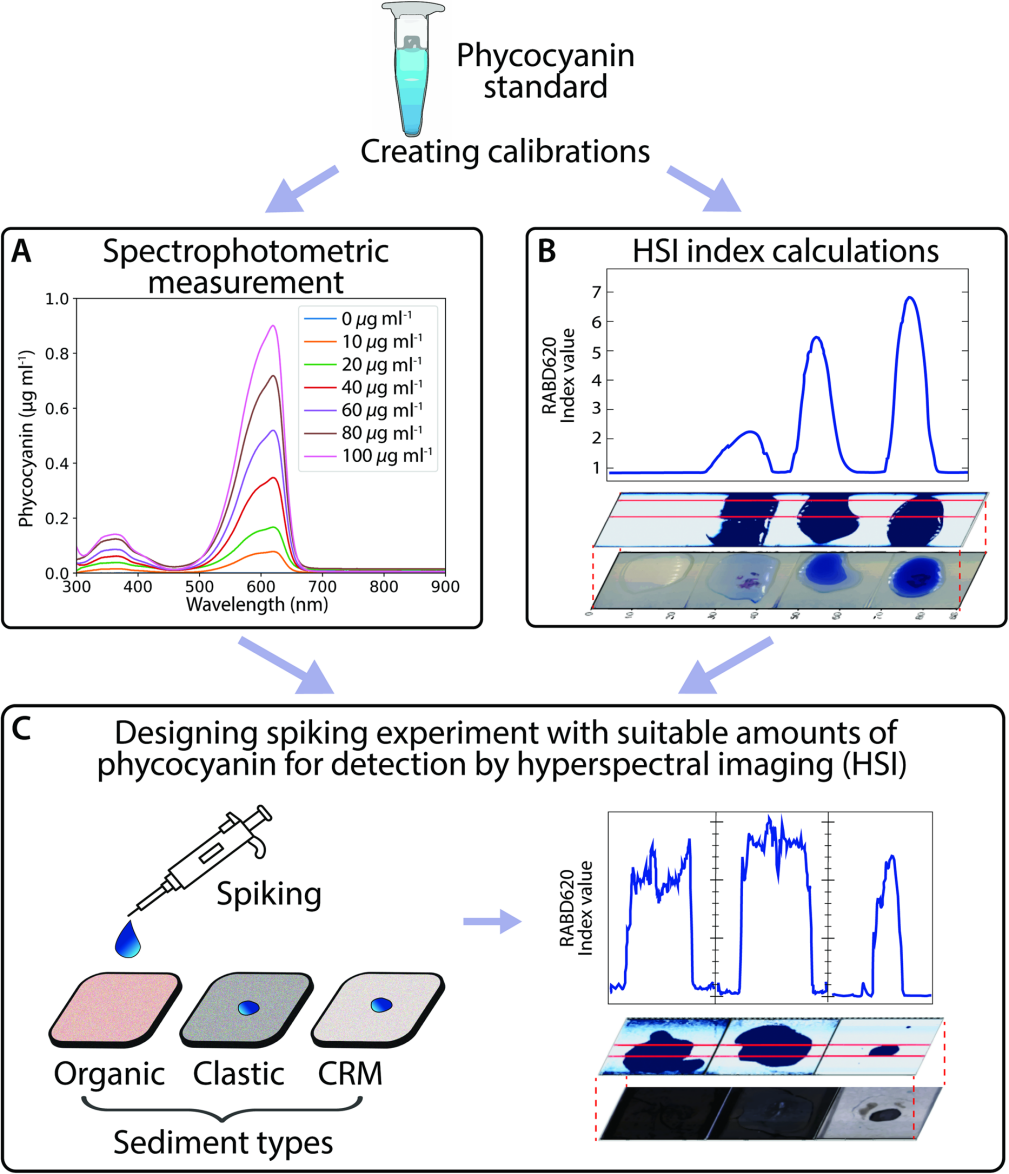
A simplified workflow diagram of our spiking experiment: Using known amounts of phycocyanin to build a calibration from (**A**) spectrophotometer data using standard solution, (**B**) Hyperspectral imaging (HSI) of microscopic slides with standard solution and (**C**) HSI of three sediment types—Organic: organic matrix, Clastic: clastic matrix, CRM: Certified Reference Material: BCR-280R. The full workflow diagram can be found in Supplementary Material Figure S1

To evaluate the hyperspectral determination of PC in lake sediments, we employed a spiking experiment (Figs. 1, S1) described below.

Two types of standards were used for the spiking experiments, (i) powder C-phycocyanin standard extracted from *Spirulina* sp. (P2172 Sigma) and (ii) liquid C-phycocyanin protein suspension in 60% ammonium sulfate, 50 mM potassium phosphate (pH 7.0) (Santa Cruz, sc-499343). We limited the addition of liquid to avoid biasing the sediment-water content and applied highly concentrated PC standards. A standard solution of PC was prepared with the powdered C-phycocyanin extracted from *Spirulina* sp. in K-phosphate buffer (KH_2_PO_4_) at pH 6.8–7.0. Calibration solutions in the range of 0 to 100 µg ml^−1^ (= 0.0–0.1 mg ml^−1^) were prepared from the standard stock solution from both types of standards. The PC concentration was determined by UV–VIS spectrophotometry using the equation by Bennett and Bogorad (1973), with the absorbance at 620 nm.

The Chl *a* standard stock solution was prepared by dissolving the Chl *a* standard powder (Sigma Aldrich C5753, Chl *a* from spinach) in 100% acetone (HPLC grade). Concentrations were calculated based on the absorbance using the Beer-Lambert equation (Eq. 1) with known extinction coefficient for 100% acetone solvent.

1 $$ A_{\lambda } = \alpha_{\lambda } \cdot L \cdot c, $$

where *A*_*λ*_ stands for absorbance, *α*_*λ*_ is the specific extinction coefficient; for Chl *a,* we used 88.15 l*· g*^*−*1^ *· cm*^*−*1^ (Jeffrey and Humphrey 1975), *L* is the optical path length and *c* is the concentration, in our case of Chl *a*.

All calibration models were calculated using ordinary least square regression conducted in R programming language (version 4.2.1, R Core Team, 2023), plotted in Python. The significance of linear regression is presented as p-values, coefficients of determination (R^2^) and the root mean square error of prediction (RMSEP), which was estimated using tenfold cross validation method.

### Sediment material

To evaluate the PC absorption feature in different sediment matrices, we selected different types of lake sediment and homogenized certified sediment-reference material (CRM) (Table 1). Sample aliquots of 1 g wet sediment weight were prepared (homogenized) and filled in 1.5 × 1.5 × 0.5 cm sample boxes. The sample surfaces were smoothed. CRM samples were dried, thus we used only 0.5 g of the sediment.

**Table 1:**
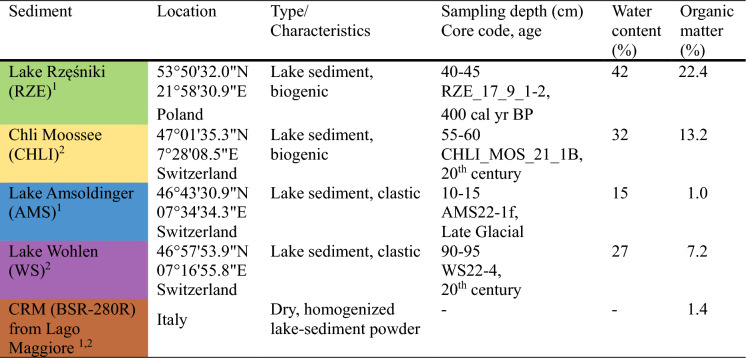
Overview of sediment used in the spiking experiment

^1^Spiking experiment 1, PC spikes reaching 0 µg, 20 µg, 30 µg and 62.6 µg of PC on sediment

^2^Spiking experiment 2, PC spikes resulting in 0 µg, 50 µg, 100 µg and 150 µg of PC on sediment, Chl *a* spikes of 0 µg, 5 µg, 10 µg, 15 µg and 20 µg of Chl *a* on sediment

*Colour code will be used in the following figures

### Spiking

Prior to spiking, all the sediments were scanned to determine the background spectra of the sediment matrices. Two separate spiking sessions were conducted using two different sets of sediment (Table 1 and Fig. S1). Due to the complex structure of the PC molecule (it is bound to phycobiliprotein, which accounts for 40–90% of the molecular weight of the PC solid standard), the actual amount of the PC varies significantly in commercial standards. This makes preparing a reliable PC stock solution challenging (for more details see the Supplementary Material). Once the stock solution concentration was determined, reliable dilutions were prepared for spiking. The use of a liquid PC standard provided a more reliable PC concentration determination, so it was used in the second spiking session.

In the first spiking session, sediments were spiked with 20 µg, 30 µg, and 62.6 µg (all standard available) of PC on 1 g of wet or dry sediment. In the second spiking session, 50 µg of PC standard was added three times to the sediment aliquots, resulting in 50 µg, 100 µg and 150 µg of PC on sediment samples. These were scanned each time.

The amounts of PC for spiking were decided based on a preceding test, where amounts below 50 µg of PC mixed into the sediment were barely detectable with the HSI. Thus, the spiking was conducted without mixing the standard solution into the sediment until the final spiking step of 62.6 µg and 150 µg of PC on 1 g wet or dry sediment.

The interference of Chl *a* on PC detection was evaluated by maintaining a constant PC concentration in the sediment sample (PC = 150 µg) and incrementally adding 5 µg aliquots of Chl *a* standard up to 20 µg. After each addition, the samples were scanned. The entire spiking was applied to the homogeneous sediment surface, without mixing the sample. At the final step of sediment spiking (PC = 150 µg, Chl *a* = 20 µg), the sediment was mixed with the spiked standards and scanned. To determine the pure standard absorption of PC, drops of the PC standard calibration solution were scanned on glass slides with white background (Fig. 1).

### Hyperspectral imaging set-up

Spiked sediments were scanned with a hyperspectral imaging scanner (Specim) equipped with a PFD-CL-65-V10E (400–1000 nm) camera. The hyperspectral data were processed utilising ENVI software following the workflow established in Butz et al. (2015). We scale our data between 0 and 1, where 1 is total reflectance (approximated with BaSO_4_ standard material) indicating minimal absorbance, and 0 represents total absorbance (dark reference = closed aperture) indicating 0 reflectance. Thus, these values can be used interchangeably. We employed two indices: (i) Relative Absorbance Band Depth (RABD, Eq. 2) and (ii) Relative Absorbance Band Area (RABA) both for PC at 620–621 nm and for total chlorins at 671–675 nm.

2 $${RABD}_{min}=\frac{\frac{{X}_{right}\bullet {R}_{left}+ {X}_{left}\bullet {R}_{right}}{{X}_{right}+{X}_{left}}}{{R}_{min}}$$

where *min* is the reflectance at the deepest point of the trough, e.g., 620 or 675 nm, *X* stands for number of bands either from the left side of the trough to the middle (*X*_*le*ft_) or from the right to the middle (*X*_*right*_), R_min_ is reflectance at selected trough minimum band/wavelength (Butz et al. 2015; Schneider et al. 2018).

To determine the absorption trough per pigment standard, the spectral profiles of calibration samples and the spiked sediment were explored. Based on the spectral profiles, we selected left and right bands as well as the trough minimum (Table S1) applicable to all our sediment types. To obtain a single value per each spiking step per sediment type, sample statistics calculating the average RABD and RABA values of the entire spiked sediment area were used. In short, the RABD and RABA is computed for all pixels and a mean value of all these pixel-specific indices is reported.

To remove the differences between the sediment types/matrices, we have normalised the RABDs with the RABD value of respective not-spiked sediments, i.e. RABD_620 (20ug spike)_/RABD_620 (raw sediment)_. Thus, all the RABD data presented are normalised this way.

### Application test on lake-sediment cores

To test the applicability of the hyperspectral RABD_620_ index as a biomarker for past cyanobacterial abundance, we scanned sediment cores from Holzmaar (50°7′8″N, 6°52′45″E, Germany; Stockhausen and Zolitschka 1999), Lago Grande di Monticchio (40°56′40″N, 15° 36′30″E, Italy; Hansen 1993), and Lake Lobsigen (47°1′50″N, 7°17′53″E, Switzerland; Ammann 1986). The hyperspectral scans of these sediment cores were processed using the established workflow, and the RABD_620_ and RABD_660_ indices were calculated.

### Preliminary extraction tests

We conducted extraction tests on sediment samples spiked with PC standard to evaluate the extraction efficiency of PC from clastic (Lake Wohlen) and organic-rich sediments (Lake Rzęśniki). We followed conventional extraction methods designed to extract PC from cyanobacteria cultures (Jaeschke et al. 2021). In the first step, the spiked sediment samples were freeze-dried and transferred to sample tubes with 2 ml purified water (Milli-Q). The samples were vortexed, sonicated and put into a centrifuge (3500 rpm/5 min). Then, 1.5 ml of the supernatant was filtered using a 0.45 µm PTFE filter and measured by UV–VIS spectrophotometry and fluorescence. No PC was detected.

In the second step, *∼*1 g aliquots of wet sediment were spiked with 1 ml PC standard solution (10 µg PC in 1 ml 50 mM K-Phosphate buffer at pH 7). The samples were stored at 4 °C for 24 h. Subsequently, the samples were vortexed, sonicated and centrifuged. MgCl_2_ was added to reduce the suspension of particles. The supernatant was filtered (0.45 µm PTFE) and measured by UV–VIS spectrophotometry and fluorescence. No PC was detected.

Lastly, 1.5 ml of 0.5 M CaCl_2_ was added to the sample, vortexed, sonicated, shaken for 3 h and stored over night at 4 °C. Subsequently, the supernatant was filtered and measured by UV–VIS spectrophotometry and fluorescence. No PC was detected.

Overall, extraction methods used for isolating PC from algal cultures or water samples exhibit low efficiency and appeared not to be suitable when applied to complex sediment matrices.

## Results

### Method validation with standard solution

**Fig. 2:**
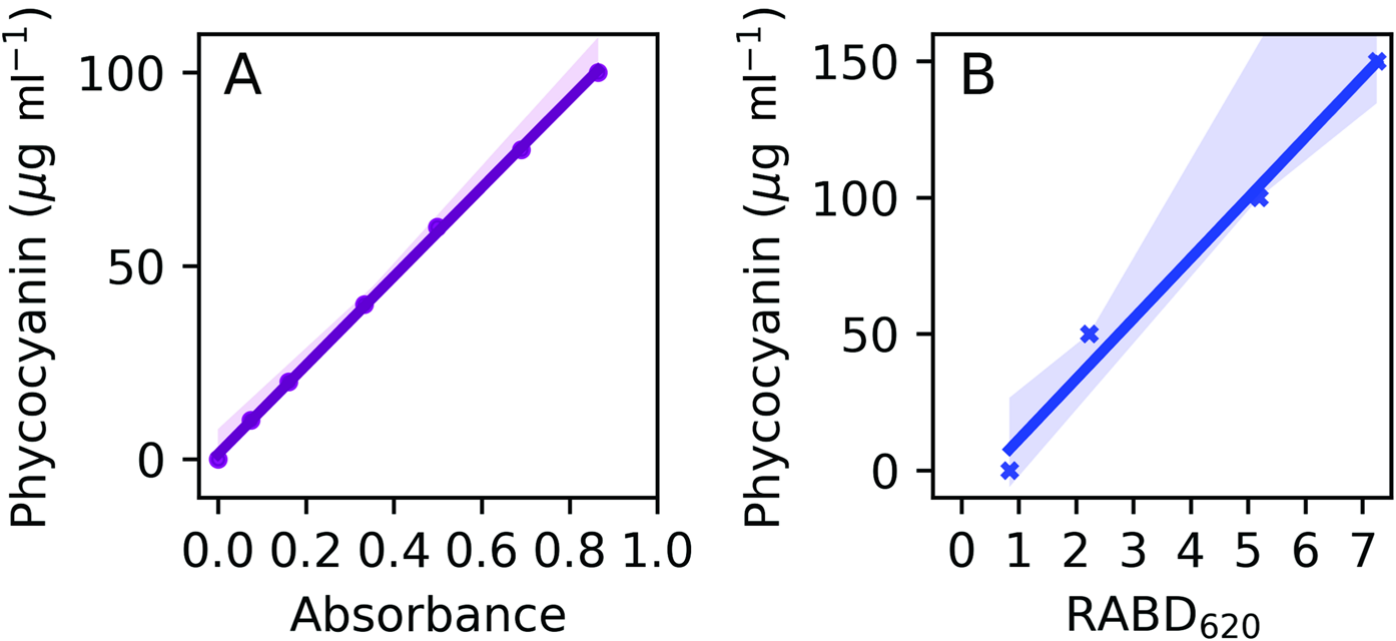
(**A**) Spectrophotometer calibration: Linear regression model between spectrophotometer absorbance and PC concentration. Shading stands for confidence interval of 100% (95% confidence interval was hardly visible). (**B**) Hyperspectral imaging calibration: Linear regression model comparing hyperspectral-derived RABDs and PC concentration. Shading stands for confidence interval of 95%

To calibrate the spectral signals with absolute PC concentrations, we computed linear regression models between the spectrophotometrically inferred absorbance of PC and its concentrations, and hyperspectral-derived RABDs and PC concentrations. The spectrophotometer calibration line of the absorbance at 620 nm and the range of concentrations from 0 to 100 μg ml^−1^ of PC standard dissolved in K-buffer shows a linear response (Fig. 2A, R^2^ = 0.9994, p-value = 2.4·10^−9^, RMSEP = 1.5%, n = 7). Similar to the spectrophotometer calibration, we compared the hyperspectral-derived RABD_620_ with absolute PC pigment amount (0–150 µg in K-buffer on microscopic slides, Fig. 2B). Also, this comparison results in a linear relationship (R^2^ = 0.97, p-value = 0.0085, RMSEP = 20%, n = 4).

### Method validation on sediment samples

**Fig. 3:**
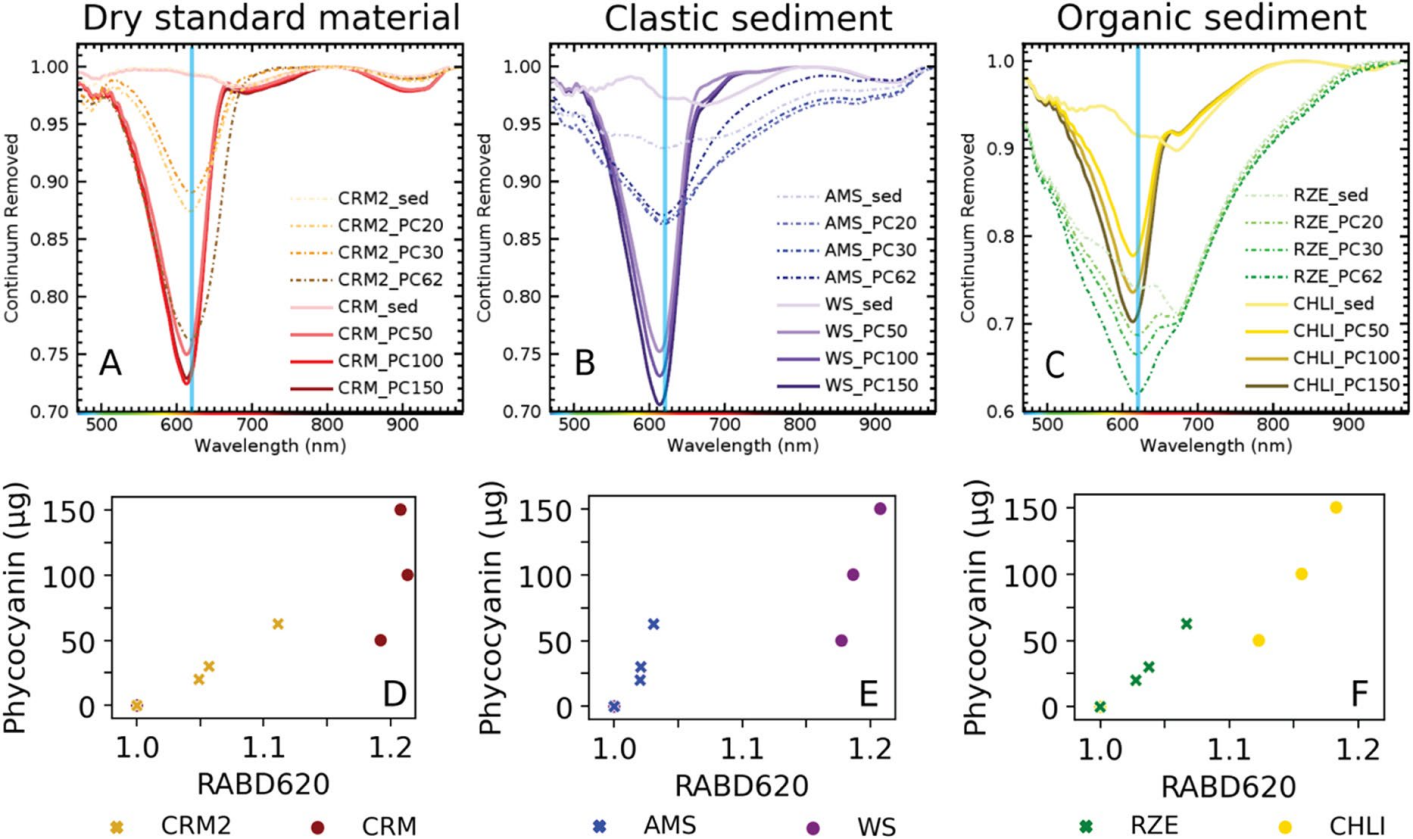
Upper panel: Mean absorbance spectra (continuum removed*) of three sediment types with increasing phycocyanin concentrations in two separate spiking experiments (Figure S1, Table 1)—experiment 1: dashed lines and crosses, experiment 2—full lines and points: (**A**) dry and homogeneous, fine-grained CRM (BSR-280R sediment from Lago Maggiore) in (CRM2 and CRM), (**B**) wet clastic sediment of Amsoldingersee (clay, AMS) and Wohlensee (sandy silt, WS) (**C**) wet organic sediment of Rzęśniki (RZE) and Chli Moossee (CHLI). Lower panel (**D**–**F**): Scatter plots presenting the relationship between the RABD_620_ and spiked phycocyanin amounts. *Continuum removal is a mathematical function to normalize reflectance spectra by the background signal unrelated to the specific absorption features. It is done by convex hull fit over the top of the spectrum using straight line segments connecting local spectra maxima (ENVI Tutorial, accessed 20/03/2024)

Subsequent to the standard-solution-spiking experiments, we investigated whether the linear relationship between the RABD_620_ and spiked PC amounts can be reproduced in different types of sediment. The sediment-spiking experiments with PC pigment concentrations from 20 to 150 µg g^−1^_wet sediment_ (22 to 292 µg g^−1^_dry sediment_) resulted consistently in hyperspectral absorption troughs at 620 nm, regardless of the sediment matrix (Fig. 3). This suggests that interference of the sediment matrix is negligible and that RABD_620_ is, indeed, diagnostically related to PC.

The spiking experiment further revealed a proportional response of the hyperspectral absorption trough deepening with higher spiked PC mass in all sediment matrices (Fig. 3). Even though the response of the RABD_620_ on the PC amount varies between sediment type (wet, dry, clastic, organic), overall correlations between PC amount and RABD_620_ including organic, clastic and reference sediments are statistically significant (n = 24, r = 0.61, r^2^ = 0.37, p < 0.001; Table S2, Fig. S2 and S3).

When inspecting the individual spiked lake sediments with very small numbers of observations (n = 3–4), the correlation is often not significant (Table S2), compared to the correlations using more data points. This can be attributed to the low number of data points. However, it is notable that 60% of all tested correlations (Table S2) are significant (p-value < 0.05).

The varying slopes and intercepts between sediment types (Fig. 3) may be explained by differences in physicochemical sediment properties, such as porosity, water content, mineralogical composition and organic matter content (matrix effect). These properties can influence the sorption of PC solutions by the sediment, which may, in turn, affect the RABD_620_ response measured at the sediment surface. This is demonstrated through individual linear regression models (Table S2, Fig. S3). While these sediment properties can impact quantification, underscoring the need for site- and sediment-specific PC calibrations, the sediment matrix has a negligible effect on the qualitative detection of PC. As a result, HSI remains a valuable tool for the rapid detection of PC.

### Interference of chlorophyll *a* with in-situ PC (RABD_620_) measurements

Since the absorption of Chl *a* is very strong and environmental concentrations are typically high, the Chl *a* hyperspectral absorption trough (RABD_675_) may interfere with the absorption trough of PC (RABD_620_) (Sorrel et al. 2021). Therefore, we additionally spiked PC-enriched sediment (PC = 150 µg) with a range of Chl *a* standard masses (5–20 µg added) to explore the potential interference of the hyperspectral absorbance features of PC and Chl *a*.

**Fig. 4:**
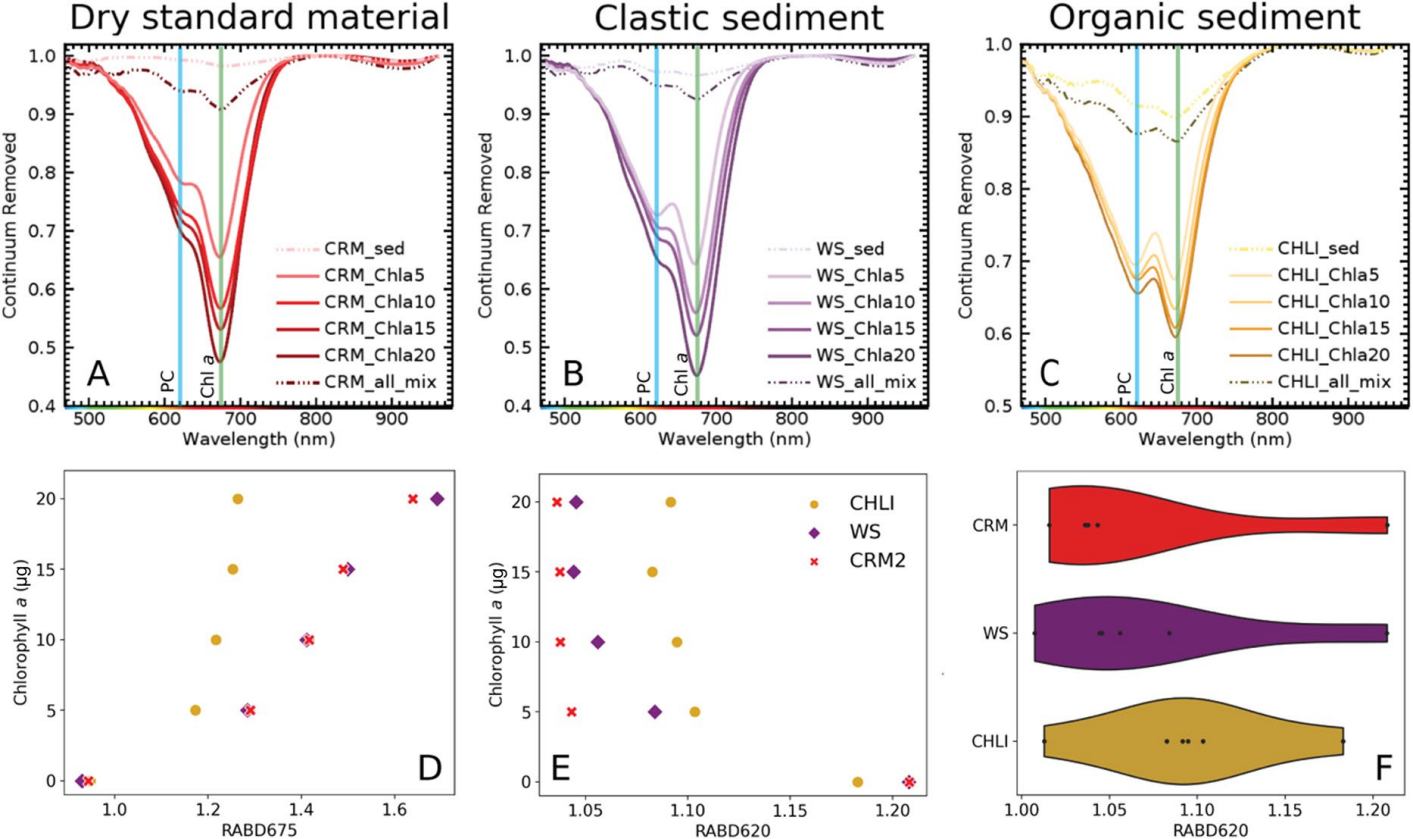
Upper panel: Mean absorbance spectra (continuum removed) of three types of PC-spiked (150 µg of PC) sediments in response to increasing Chl *a* spiking: (**A**) dry and homogenous, fine-grained CRM (BSR-280R sediment from Lago Maggiore), (**B**) wet clastic sediment of Lake Wohlen (WS), (**C**) wet organic sediment of Chli Moossee (CHLI). Lower panel: (**D**) Response of the spectral index RABD_675_ on spiked Chl *a* concentration, (**E**) Response of the spectral index RABD_620_ on Chl *a* spiking. (**F**) Distribution of the spectral index RABD_620_ values calculated for each Chl *a* spiking step (0–20 µg Chl *a*), while maintaining PC concentration constant (150 µg of PC). This demonstrates the effect of Chl *a* on the phycocyanin index RABD_620_

In the hyperspectral imaging data, we observe a trough at ~ 675 nm associated with the Chl *a* standard (Fig. 4A–C). Similar to the PC spiking, a deepening of the absorbance trough as a response to the increasing mass of Chl *a* in the sediment is observed in all types of sediment matrices (Fig. 4A–C) and results in close-to-linear relationships (Fig. 4D).

We also observe a response of the absorption at 620 nm and thus the RABD_620_ on the added Chl *a* standard, even though the PC amount in the sediment is kept constant (150 µg, Fig. 4A–C and F). Therefore, we have tested the linearity of this response using linear regression models (Table S3). We observe negative relationship between Chl *a* and RABD_620_ (Fig. 4D), which results in underestimating the PC concentrations with increasing Chl *a* concentration. Additionally, the ratio between Chl *a* and PC concentration in the sediment seems to play an important role in PC detection.

Small concentrations of Chl *a* (as low as 5 µg of Chl *a* g^−1^_wet sediment_ or per 0.5 g_dry sediment_) resulted in RABD_675_ values similar to the RABD_620_ values in Chl *a-*free sediment with 150 µg PC. This highlights the strong light-absorbance efficiency of Chl *a* (~ 0.55 continuum removed absorbance trough depth at 20 µg of Chl *a* compared to ~ 0.8 at 20 µg of PC) compared to the PC. Our spiking experiment revealed that as the ratio of Chl *a* to PC increases, the RABD_620_/RABD_675_ ratio decreases, suggesting that a larger Chl *a* trough can mask the PC trough (Fig. S4A). At a Chl *a*/PC ratio of 0.13 (20 µg/150 µg), both troughs are distinguishable, with an RABD_620_/RABD_675_ ratio of 0.61. However, as the Chl *a*/PC ratio increases, the separation between the troughs diminishes, as indicated by lower RABD ratios. Our current spiking setup does not fully capture the extent of Chl *a*'s interference with the PC trough.

Moreover, the extent of the potential effect of Chl *a* absorbance on the quantification of PC may change depending on the approach used to determine the RABD index. When calculating the RABD index, the selection of wavelengths defining the beginning (left) and the end (right) of the trough directly affects the RABD values (Eq. 2). The quantification becomes even more complex when using absorption trough areas. We have used a conservative, non-overlapping approach (Table S1), which likely underestimates the trough depth and thus the RABD index value but ensures that the RABD_620_ represents PC only and not a mixed signal with Chl *a*. A deconvolution approach (Sanchini and Grosjean 2020) could help for more accurate RABD_620_ determination and, thus, PC quantification.

Even though we have demonstrated that the presence of Chl *a* in the sediment brings uncertainties in the PC quantification, we also show that the qualitative detection of PC is still possible.

### Phycocyanin (RABD_620_) in lake sediments: examples from European lakes

**Fig. 5:**
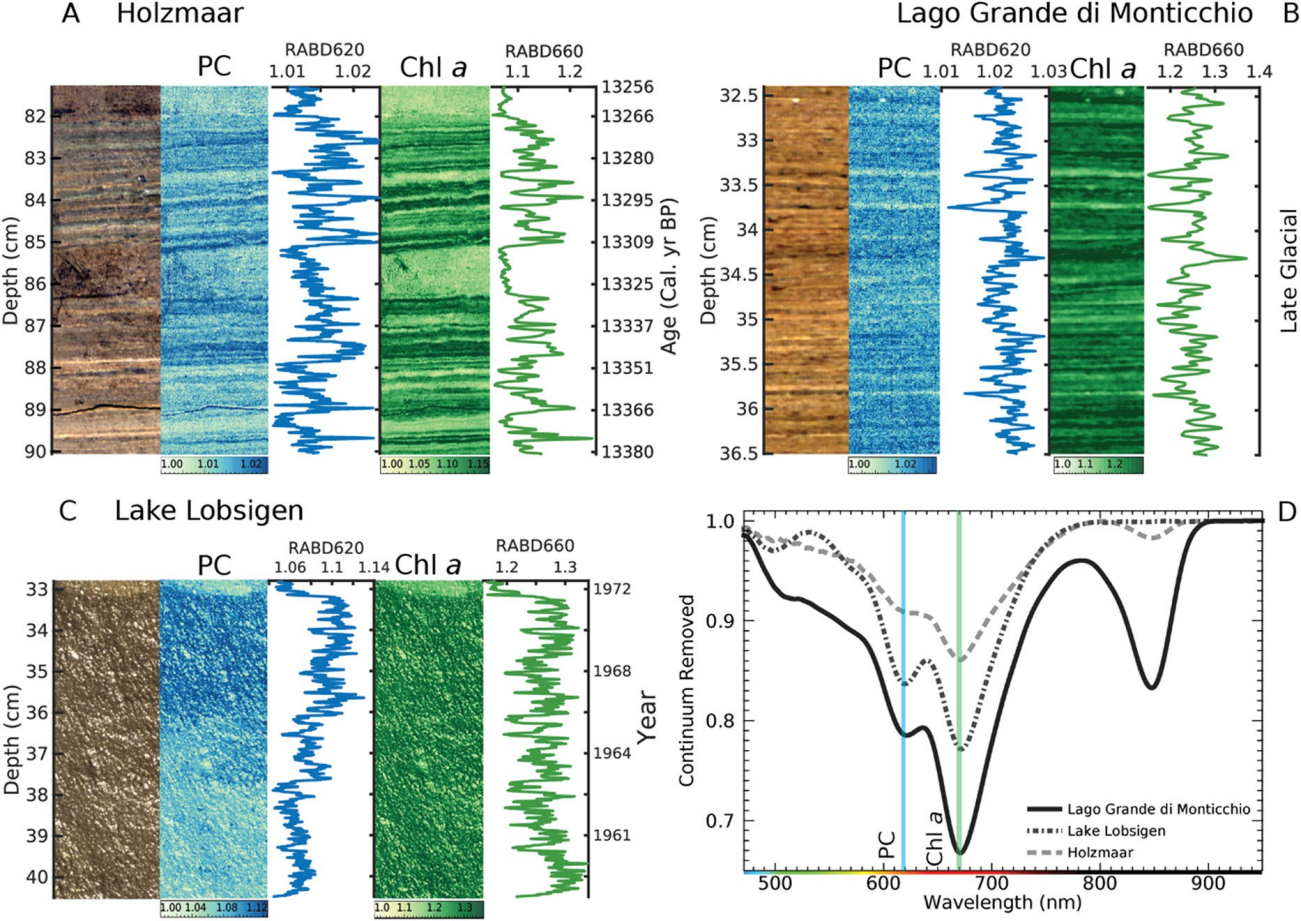
An example close-up on sediments: (**A**) Holzmaar, (**B**) Lago Grande di Monticchio and (**C**) Lake Lobsigen including core photo (left, RGB true color), hyperspectral intensity maps and time series of RABD_620_ (PC, blue, middle) and RABD_660_ (Chl *a; g*reen, right). Age-depth models are available only for Holzmaar (Birlo et al. 2023) and Lake Lobsigen (Schaad 2022). (**D**) Average spectral profiles (continuum removed) of all three example lakes

We applied the hyperspectral RABD_620_ index to sediments from Holzmaar (Germany), Lago Grande di Monticchio (Italy), and Lake Lobsigen (Switzerland) to trace the past occurrence of cyanobacteria. Sediments of Holzmaar and Lago Grande di Monticchio (sediment sections of 8 and 4 cm, respectively, both late glacial in age) are examples with a laminated structure (varves) and seasonal cycles of Chl *a* (RABD_660_) and PC (RABD_620_) (Figs. 5A, B). With the hyperspectral scanning approach, we are thus obtaining relative changes in both, Chl *a* and PC on a sub-millimeter scale (pixel size ∼80 µm), which can be used for detecting seasonal changes in lake productivity, depending on the sediment-accumulation rate.

In case of Lake Lobsigen, sediments (1960–1972 CE) are not laminated due to massive bioturbation. Here, we detect prominent PC occurrence above 38–36 cm sediment depth (corresponding to 1964 CE) in a period when the lake was eutrophic/hypertrophic. Here, the Chl *a*/PC ratio is relatively low compared to the two other lakes (Figs. 5C, D). Therefore, the PC trough is very well distinguishable from the usually more dominant Chl *a*/PC absorbance trough.

The spectra from Lago Grande di Monticchio and Holzmaar (Fig. 5D) also show an absorption trough at 840 nm which corresponds to bacteriopheophytin *a*, a pigment diagnostic for purple sulfur bacteria and the presence of a chemocline in the photic zone (Butz et al. 2015; Zander et al. 2023).

## Discussion

Our experiment demonstrates that HSI is a powerful technique to directly (in situ) detect the photopigment PC in lake sediments. The absorbance trough was identified at 620 nm and its depth is proportional to the mass/concentration of PC in the sediment. Therefore, the HSI approach offers a solution to the challenging wet chemical methods of PC extraction. Our study further reveals detection differences in the RABD_620_ response to varying sediment matrices and properties. Furthermore, we observe a spectral interference with Chl *a*. Subsequently, we discuss these variations and highlight implications for the application of HSI for PC detection in lake sediments.

### Sediment matrix

Our spiking experiment has shown that the sediment matrix does play a role in the *in-situ* PC quantification. Importantly, the best linear relation between spiking (mass/concentration) and HSI index RABD_620_ values is found in organic sediments, where eutrophication and cyanobacterial blooms are expected first. The regression line is very steep in clastic and freeze-dried sediments where the HSI detection of PC seems more problematic. We mostly consider two confounding factors: (i) differences in the water content and porosity/grain size of the sediment: we are unable to estimate the amount of the spiked PC mass remaining on the sediment surface (visible for HSI) and the amount of PC mass disappearing in the pores (invisible for HSI); (ii) PC-preservation: the chemical interaction of PC with the sediments and its preservation still remains unknown, and thus, also the understanding of how PC is ‘visible’ by HSI or extractable by wet chemical methods.

### Spectral interference of chlorophyll *a* with phycocyanin

As demonstrated in the spectral profiles (Figs. 4), quantification of PC in sediment remains challenging due to the spectral overlap with its green counterpart, Chl *a*. This overlap in the range of 600–700 nm creates a deceptive masking effect (Yacobi et al. 2015; Lauceri et al. 2017; Sorrel et al. 2021), often leading to limited or no detection of PC, and overestimating of Chl *a*. In cases where Chl *a* concentrations are significantly higher than PC, HSI-inferred PC detection becomes unreliable or even completely masked, as indicated by the negative correlation between the RABD ratios and the Chl *a*/PC concentration ratio (Fig. S4A). To better understand this masking effect, future research should design experiments where PC concentration is kept constant while Chl *a* concentration is gradually increased until the PC trough disappears. This approach could help establish a threshold for when this masking phenomenon occurs.

The complexity of this challenge lies in the inherent coexistence of PC and Chl *a* within cyanobacteria, alongside varying carotenoids, which has been previously investigated by Favot et al. (2020). The ratio of Chl *a* and PC in cyanobacteria is, however, highly variable depending on the growth phase, species and nutrient availability (Foy 1993; Randolph et al. 2008; Macário et al. 2015; Rousso et al. 2022). Recent studies have revealed that the mere presence of Chl *a* can overestimate PC amounts by up to 78.8% in spectrometric techniques, and various correction methods and algorithms were suggested (Simis et al. 2005; Le et al. 2011; Lauceri et al. 2017; Dev et al. 2022).

Furthermore, pigment degradation in sedimentary archives may affect the ratio of Chl *a* and PC. Chl *a* is expected to degrade faster than PC in vitro, as evidenced by observations made on our stock solutions during our spiking experiments. Nevertheless, several studies suggest that Chl *a* is relatively stable and for hundreds of years well-detectable after deposition in the sediments (Rydberg et al. 2020). Therefore, the present data suggest that the Chl *a* signal from HSI should not be heavily affected by degradation processes. However, there is still a lack of understanding of the degradation rates of PC in different sediment matrices.

Therefore, we would not advise using the ratio of Chl *a* and PC in sediments to constrain past cyanobacterial production. Instead, we suggest focussing on relative changes in the RABD_620_ index when the Chl *a*/PC ratio is low (0–0.2; Fig. S4A) or when Chl *a* concentrations are below 300 µg g^−1^ wet sediment, values commonly observed in natural sediments (Butz et al. 2017; Makri et al. 2021; Zander et al. 2023).

### Numerical calculation of the absorbance trough

The optimal methodology for evaluating the spectral signal of sedimentary pigments remains a subject of discussion. Proposed approaches include the use of total trough area, ratios, relative trough band depth or area, as outlined by Ghanbari et al. (2023), and orthogonal partial least squares regression modelling, as demonstrated by Meyer-Jacob et al. (2017) and Trygg and Wold (2002). We have used a conservative approach of relative absorbance band depth (RABD) and tested for the relative absorbance band area (RABA), while defining the left and right limits of the absorbance trough (Table S1). To us, the best representing index remained the RABD with the narrow ranges of the trough limits. In this way, we may underestimate the absolute trough depth, but we avoid the interference with the Chl *a* trough for PC and vice versa.

In the remote sensing realm and spectroscopic literature, various algorithms for spectral signal deconvolution are suggested (Thrane et al. 2015; Brossard et al. 2016; Sanchini and Grosjean 2020; Zieger et al. 2020), which may help to diagnose further pigments related to algal/bacterial groups, e.g. green sulphur bacteria pigments at 715 nm (Zander et al. 2023) or the PC (610–620 nm, this study).

### Current limitations in sedimentary phycocyanin

While laboratory studies have provided valuable insights into the PC producers, metabolism and phycobilisome structure (Gray et al. 1973; Gantt 1980; Zilinskas and Greenwald 1986; Sidler 1994; Takano et al. 1995; Schluchter and Glazer 1999; Padyana et al. 2001; Patel et al. 2005; Khattar et al. 2015), its natural abundance in lake sediments remains largely unknown. The encapsulation within proteins could provide stability and protection from environmental stressors, but on the other hand, it makes extraction and analysis of PC from sediments more difficult or even impossible. Additionally, the fate of PC within sediment matrices remains poorly understood, limiting our ability to fully comprehend its stability and preservation in sedimentary archives.

At least two key factors limit our understanding of sedimentary PC: (1) the complexity of extracting it from sediments, and (2) the lack of knowledge on how and in what form (entire phycobilisome or isolated PC) it is preserved within the sediment matrix. However, understanding the specific mechanisms of PC preservation would be crucial for accurately evaluating its stability and interpreting its presence in the sedimentary record.

Therefore, the development of more sensitive and effective methods for detecting and quantifying PC *in-situ* is essential to address these knowledge gaps and gain a comprehensive understanding of its ecology and potential impact on lake systems. Hence, here we presented the first validation of the potential detection of PC in sediment matrices using the spiking experiment, which serves us an important baseline for future research focused on the quantification of PC concentrations based on HSI.

## Conclusions

Our study investigated the potential of *in-situ* hyperspectral imaging detection and quantification of the cyanobacterial pigment PC in different sediment matrices at its hypothesized absorption trough at 620 nm. We conclude our results in four major points:

- The trough at 620 nm observed in sediment is PC: We have shown that the absorbance trough at 620 nm identified in the hyperspectral scanning data of sediment cores is associated with the cyanobacterial blue pigment PC.
- The absorption trough depth is linearly proportional to concentration/amount: Further, we have shown that the depth of the absorbance trough is in a close-to-linear relationship with the concentration of PC in the sediment.
- The sediment matrix matters: Based on our spiking set-up, the most consistent linear regression was observed in organic sediments. The response of the RABD_620_ on the PC amounts in clastic sediment was much less pronounced and differing between sediments, likely due to various grain size. This makes comparing PC between lakes challenging, but the variation of RABD_620_ within individual lakes/sediment types reflects the relative changes in PC concentrations.
- Chl *a* interferes with the PC trough: As cyanobacteria produce both green and blue pigments, we have shown that there is an intricate interplay of the absorption troughs of PC (610–620 nm) and Chl *a* (660–670 nm).

Future research, including experimental set-ups, should focus on developing correction models to better constrain and minimise confounding effects of the sediment matrix and the spectral interference of Chl *a* with PC. Additionally, enhancing our understanding of PC preservation in sediments is crucial. Ultimately, this will enable us to quantify PC concentrations using HSI.

## Supplementary Information

Below is the link to the electronic supplementary material.

Supplementary file 1 (PDF 272 kb)

Supplementary file 2 (DOCX 1445 kb)

## Data Availability

All data used in this publication are available at Zenodo: Dataset: 10.5281/zenodo.13360564 (DOI) Dataset: 10.5281/zenodo.13347818 (DOI) Dataset: 10.5281/zenodo.13364569 (DOI) Dataset: 10.5281/zenodo.13364577 (DOI) Dataset: 10.5281/zenodo.13364579 (DOI) Dataset: 10.5281/zenodo.13364583 (DOI) Scripts for all the plots and statistics are available at Renku platform (https://renkulab.io/projects/petra.zahajska/phycocyanin).
