# Supplementary figures and images for: Direct detection of phycocyanin in sediments by hyperspectral imaging
Source: J Paleolimnol. 2024 Dec 30;73(1):73–87. doi: 10.1007/s10933-024-00350-y (PMC11742344; doi:10.1007/s10933-024-00350-y)

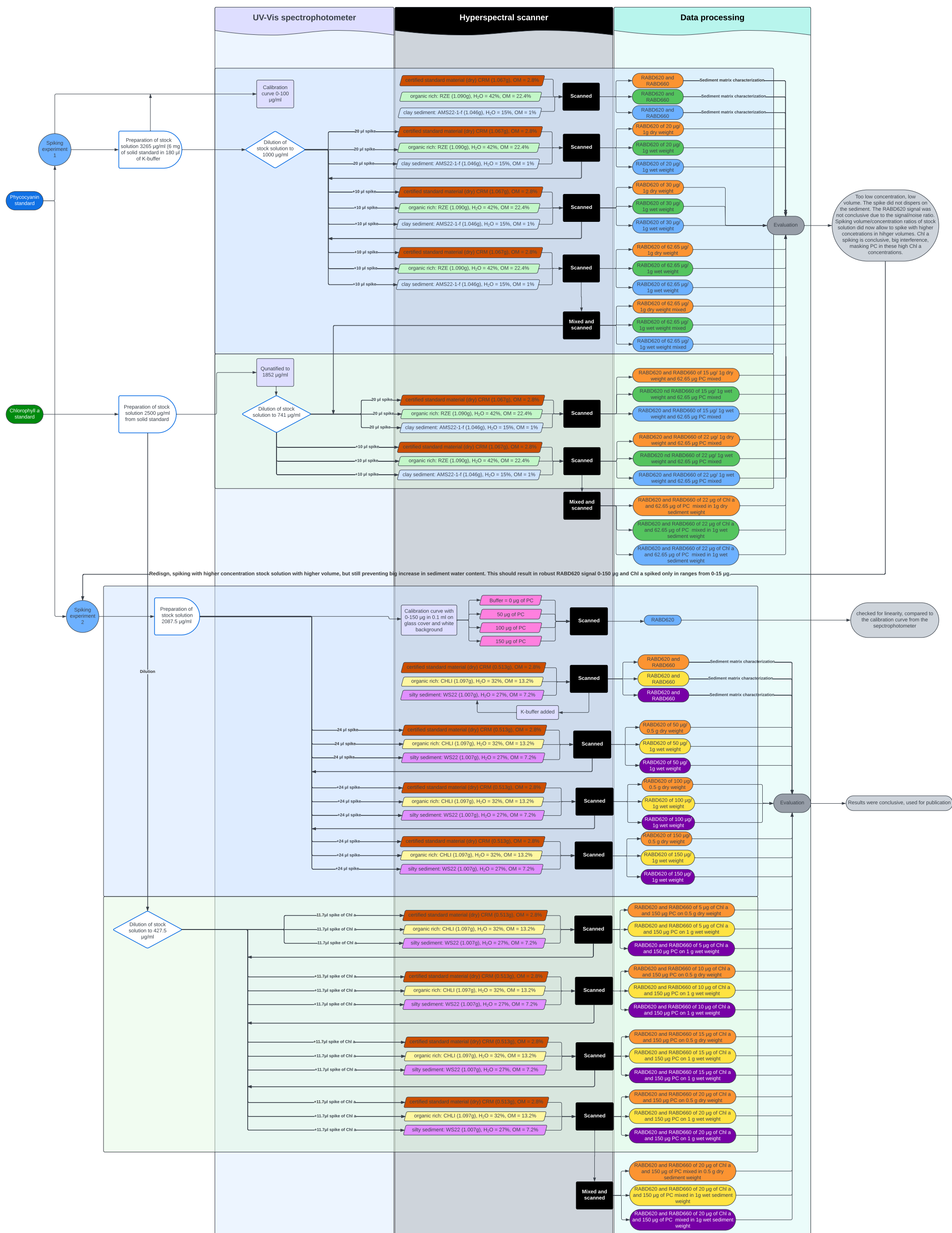

Supplement: Supplementary file 1 — (PDF 272 kb) [file 10933_2024_350_MOESM1_ESM.pdf]
