# Supplementary material for: Direct detection of phycocyanin in sediments by hyperspectral imaging
Source: J Paleolimnol. 2024 Dec 30;73(1):73–87. doi: 10.1007/s10933-024-00350-y (PMC11742344; doi:10.1007/s10933-024-00350-y)
Supplement: Supplementary file 2 — (DOCX 1445 kb) [file 10933_2024_350_MOESM2_ESM.docx]

Supplementary information


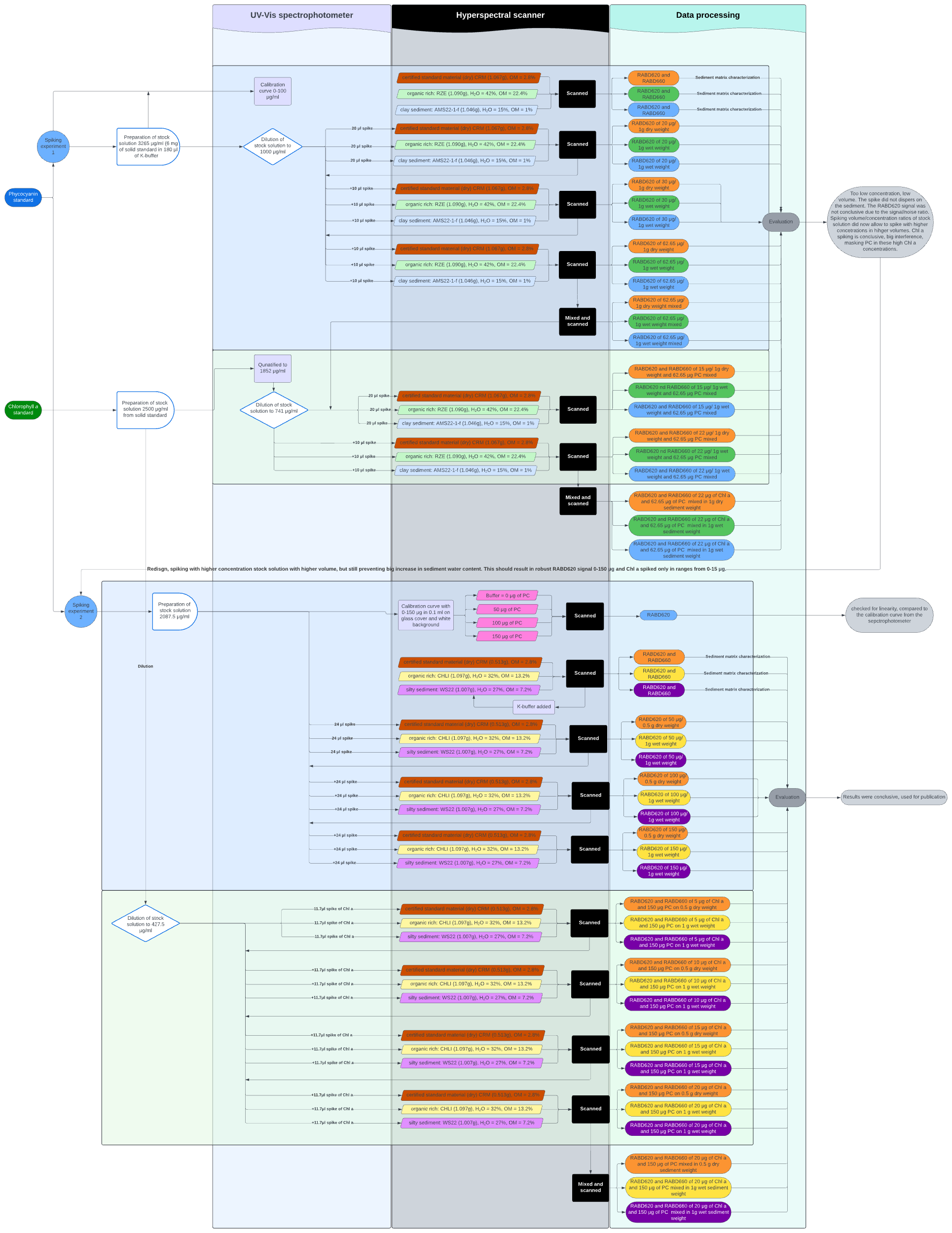


**Figure S1** Detailed spiking workflow. Also available in a separate PDF file of the supplementary material.

**Challenges in PC standard stock solution for spiking experiments**

The contribution of the protein molecular weight in the standard varied from 94-99.3% (the color substance was only 6-8.7% of the dissolved weight), it was not possible to prepare a target concentration of the stock standard solution from the solid standard. Therefore, a liquid standard with known concentration was used to construct our calibration curve, and based on this cure, we could calculate back the concentration of our stock solution used then for dilutions for spiking. It is important to note that even the liquid standard did not have the concentration stated in the standard specification sheet (20mg/ml) but instead only 75% of the stated concentration (15 mg/ml).

**Table S1** Parameters used for RABD index calculations and quantification of phycocyanin and chlorophyll *a* absorption.

| Index | Left band (nm) | Max. absorption (nm) | Right band (nm) | Applied on samples |
| --- | --- | --- | --- | --- |
| RABD_621_ | 590 | 621 | 643 | CRM2, AMS, RZE |
| RABD_671_ | 643 | 671 | 760 | CRM2, AMS, RZE |
| RABA_621_ | 590 | – | 643 | CRM2, AMS, RZE |
| RABA_671_ | 643 | – | 760 | CRM2, AMS, RZE |
| RABD_620_ | 580 | 620 | 645 | CRM, WS, CHLI |
| RABD_675_ | 645 | 675 | 780 | CRM, WS, CHLI |
| RABA_620_ | 580 | – | 645 | CRM, WS, CHLI |
| RABA_675_ | 645 | – | 780 | CRM, WS, CHLI |

**Table S2** The statistical information on linear regression models of RABD_620_ to phycocyanin concentrations for various types of sediment. In some cases, we computed both regressions, including the sediment signal, PC=0, and excluding this datapoint, as the linearity often improves by removing the sediment signal.

| Sediment type | index | p-value | R^2^ | RMSEP | equation | n |
| --- | --- | --- | --- | --- | --- | --- |
| Dry CRM | CRM2 | 0.002 | 0.99 | 6.11% | y = 0.007x + 0.99 | 4 |
|  | CRM | 0.189 | 0.49 |  | y = 0.0013x + 1.06 | 4 (3) |
|  | CRM2 + CRM | 0.14 | 0.19 |  | y = 0.001x + 1.10 | 8 |
| Wet clastic sediment | AMS | 0.046 | 0.86 | 33.68% | y = 0.002x+1.02 | 4 |
|  | WS | 0.15 | 0.58 (0.9) |  | y = 0.0012x + 1.05 | 4 (3) |
|  | AMS + WS | 0.003  (0.03) | 0.76  (0.68) | 20.83%  25.84% | y = 0.001x + 1.04  (y = 0.001x + 1.08) | 8 (6) |
| Wet organic sediment | RZE | 0.001 | 0.997 | 4.68% | y = 0.003x + 1.004 | 4 |
|  | CHLI | 0.07 (0.039) | 0.8  0.99 | 56.06%  (9.38%) | y =0.001x + 1.028  (y = 0.0006x + 1.094) | 4 (3) |
|  | RZE+CHLI | 0.013  (0.11) | 0.62  (0.39) | 25.99%  (40.4%) | y = 0.0012x + 1.04  (y = 0.0007x + 1.09) | 8 (6) |
| All |  | 0.00098 | 0.37 | 21.79% | y = 0.0013x + 1.061 | 24 |

**
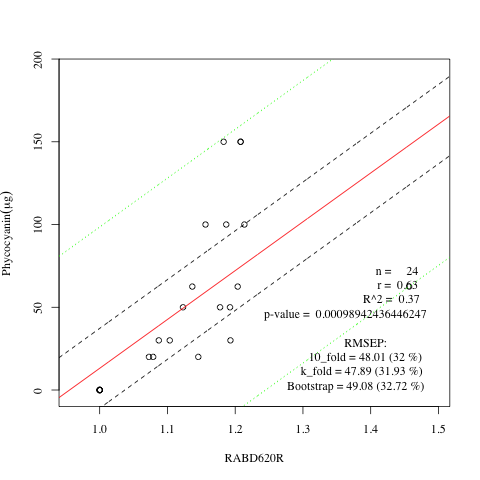
**

Figure S2: Linear regression model of all spiked samples by phycocyanin, showing the statistical information from table S2, last line.


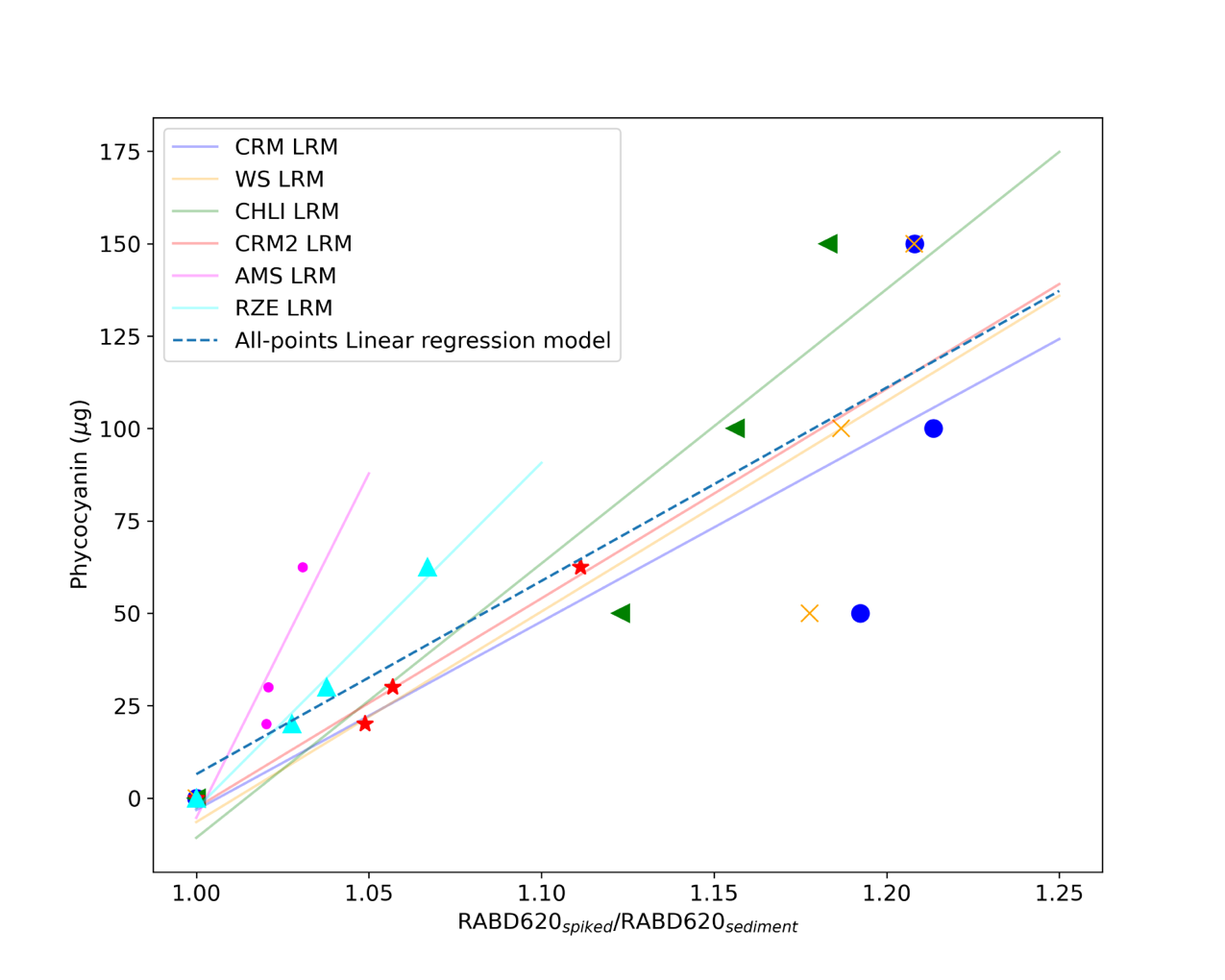


Figure S3: An overview plot of all samples spiked with phycocyanin demonstrating the various linear regression models’ slopes, likely dependent on the sediment’s physical and chemical properties. The linear regressions include the unspiked sediment values as the starting value. Slopes without this starting point, including only the values of spiked sediment would vary even more.

**Table S3** The statistical information on linear regression models of RABD_675_/RABD_620_ concentration of Chl *a* for various types of sediment. * number of samples 36 is including the sediment background, Chl *a* = 0 *µ*g

| Sediment type | index | p-value | R^2^ | RMSEP | equation | n |
| --- | --- | --- | --- | --- | --- | --- |
| Dry CRM | RABD675 | 0.008 | 0.98 | 8.7% | y = 0.02x + 1.18 | 4 |
|  | RABD620 | 0.12 | 0.67 | 87% | y = -0.0004 + 1.044 | 4 |
| Clastic WS | RABD675 | 0.012 | 0.96 | 14% | y = 0.026x + 1.145 | 4 |
|  | RABD620 | 0.11 | 0.69 | 48% | y = - 0.0025x + 1.09 | 4 |
| Organic CHLI | RABD675 | 0.03 | 0.92 | 20% | y = 0.026x + 1.145 | 4 |
|  | RABD620 | 0.28 | 0.27 | 56% | y = - 0.0035x + 1.089 | 4 |
| All | RABD675 | 0.026  (3.51 • 10^-15^) | 0.34  (0.84) | (33.3%)  (14%) | y = 0.018x + 1.16  (y = 0.03x+0.99) | 12  (36)* |
|  | RABD620 | 0.35  (0.052) | 0  0.08 |  |  | 12  (36)* |


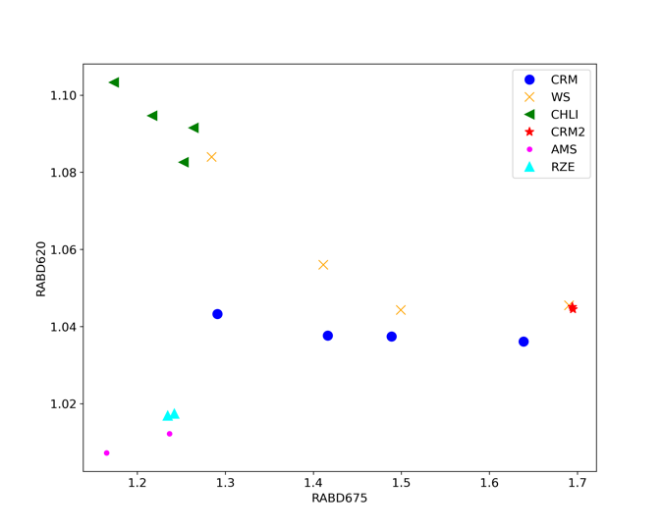

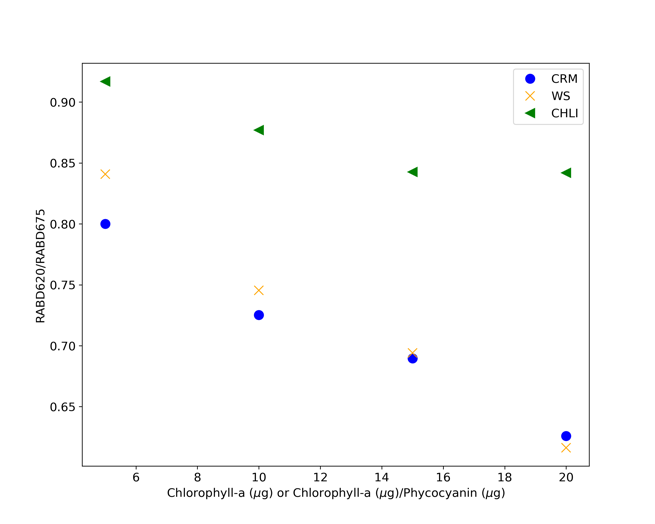

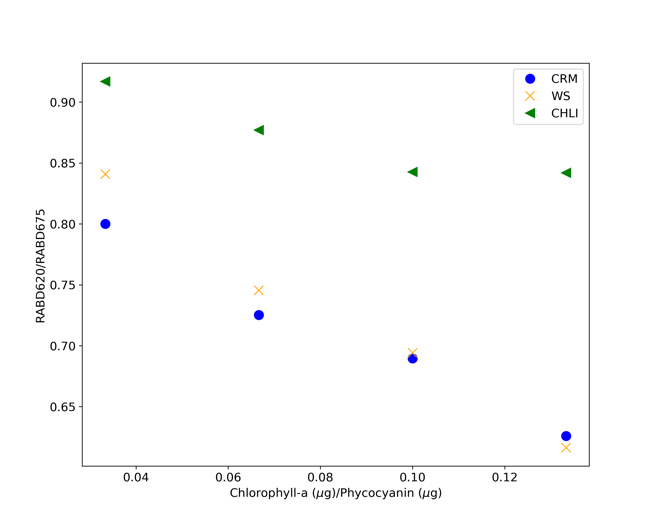


A

B

Figure S4: A) Cross-correlation plot of the ratio of RABD620/RABD675 indices and Chl *a* concentration displaying the negative correlation between these two. Similar results occur if we use the the ratio of Chl *a*/PC concentration on the x-axis, as the PC concentration during our Chl *a* spiking was kept constant. A higher concentration of Chl *a* results in lower ratios of RABDs. This implies that the pigment absorption troughs become less separable until, eventually, the PC trough would be entirely overprinted by the chlorophyll trough, when the RABDs ratio is very close to 0. B) demonstrates no significant correlation between RABD620 and RABD675 in the Chl *a* spiked sediments; According to our experiment design and relatively limited dataset, there is no straightforward method for correcting the masking effect of the Chl *a* trough on the PC trough.
